# High-Resolution Crystal Structures Elucidate the Molecular Basis of Cholera Blood Group Dependence

**DOI:** 10.1371/journal.ppat.1005567

**Published:** 2016-04-15

**Authors:** Julie Elisabeth Heggelund, Daniel Burschowsky, Victoria Ariel Bjørnestad, Vesna Hodnik, Gregor Anderluh, Ute Krengel

**Affiliations:** 1 Department of Chemistry, University of Oslo, Blindern, Norway; 2 Department of Biology, Biotechnical Faculty, University of Ljubljana, Ljubljana, Slovenia; 3 The National Institute of Chemistry, Ljubljana, Slovenia; Northwestern University, Feinberg School of Medicine, UNITED STATES

## Abstract

Cholera is the prime example of blood-group-dependent diseases, with individuals of blood group O experiencing the most severe symptoms. The cholera toxin is the main suspect to cause this relationship. We report the high-resolution crystal structures (1.1–1.6 Å) of the native cholera toxin B-pentamer for both classical and El Tor biotypes, in complexes with relevant blood group determinants and a fragment of its primary receptor, the GM1 ganglioside. The blood group A determinant binds in the opposite orientation compared to previously published structures of the cholera toxin, whereas the blood group H determinant, characteristic of blood group O, binds in both orientations. H-determinants bind with higher affinity than A-determinants, as shown by surface plasmon resonance. Together, these findings suggest why blood group O is a risk factor for severe cholera.

## Author Summary

Cholera is a severe diarrheal disease that kills a hundred thousand people per year. With climate change, the number of cases is predicted to increase to millions. Individuals with blood group O are particularly at risk. Here we report high-resolution crystal structures of the native cholera toxin of both major biotypes, in complexes with relevant blood group determinants. These structures, in combination with quantitative binding data, shed light on cholera blood group dependence. Understanding the molecular basis of this association is expected to be of considerable importance, for example for developing new vaccination strategies that take this information into account.

## Introduction

Cholera is a severe diarrheal disease caused by the pathogen *Vibrio cholerae* [1]. It originated in the Ganges delta, but is now widespread across the world. Caused by contaminated drinking water, severe cholera outbreaks often occur in war zones or in the aftermath of natural disasters. Well remembered is the recent outbreak in Haiti following the earthquake in 2010 –the worst in recent history. Every year, several million people fall ill from cholera and one hundred thousand die [2]. Individuals with blood group O have a particularly high risk of severe symptoms and hospitalization [3–8], but the molecular causes of this association are not well understood. However, individuals with blood group O are not more susceptible to cholera infection than those with other blood groups, pointing towards the critical role of cholera virulence factors, rather than bacterial colonization [3, 4].

*V*. *cholerae* is a gram-negative bacterium capable of colonizing the human small intestine. In response to environmental stimuli, the bacterium produces and secretes its main virulence factor, the cholera toxin (CT) [9]. CT is an AB_5_ protein toxin, which consists of one toxic A-subunit (CTA) and five identical B-subunits arranged in a doughnut-shaped ring (CTB) [10]. The B-pentamer itself is non-toxic and, in fact, part of a well-known vaccine (Dukoral). It is responsible for binding to the host cell receptors as a first step of pathogenesis. The primary receptor of the cholera toxin is the GM1 ganglioside, which is expressed in distinct membrane microdomains of small-intestinal epithelial cells [11, 12]. CTB can bind five GM1 gangliosides simultaneously, resulting in one of the strongest carbohydrate-protein interactions known [13]. After receptor binding, the toxin is internalized by endocytosis, where it hijacks the cells’ endogenous pathways, ultimately inducing the watery diarrhea that is characteristic of cholera [14].

There are two major biotypes of *V*. *cholerae*, classical and El Tor, which produce cholera toxins with a slight sequence variation (cCT and ET CT) [15, 16]. cCT and ET CT differ in only two residues of the B-subunit, His18/Tyr18 and Thr47/Ile47 (classical/El Tor). El Tor *V*. *cholerae* is reported to have the strongest blood group association [17]. This biotype has been dominating the current pandemic, which has been ongoing since 1961. However, since 2001 a new hybrid biotype has taken over, which has the biochemical characteristics of El Tor *V*. *cholerae*, but produces a cholera toxin with the classical sequence [18].

**Fig 1:**
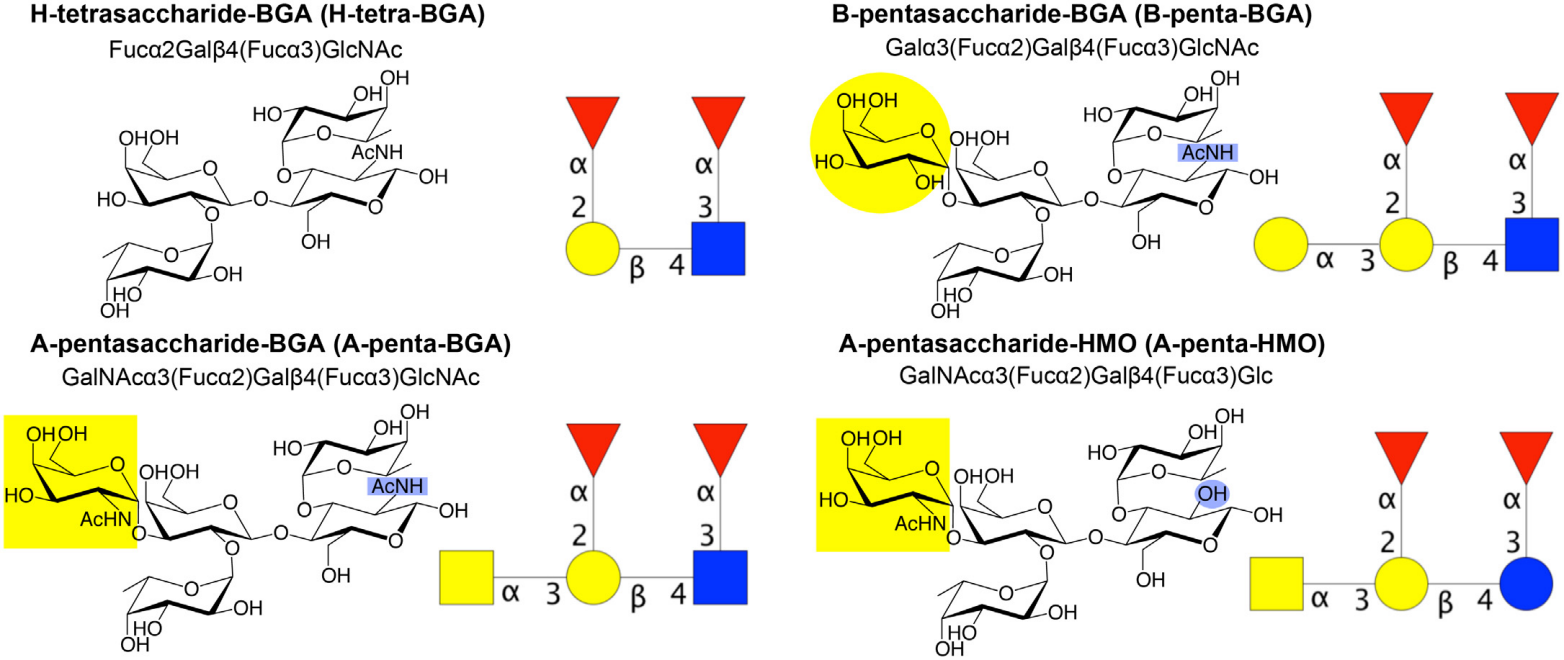
Structures and nomenclatures of the oligosaccharides in this article. Lewis-y blood group determinants H-tetra-BGA, B-penta-BGA, A-penta-BGA and the related human milk oligosaccharide A-penta-HMO. All of these have type-2 core structures. Note that H-tetra-HMO and A-penta-HMO were referred to as H-tetra and A-penta in our previous publications [27, 29]. Carbohydrate symbols follow the nomenclature of the Consortium for Functional Glycomics (Nomenclature Committee, Consortium for Functional Glycomics (functionalglycomics.org/static/consortium/Nomenclature.shtml); d-galactose (Gal)–yellow circle, *N*-acetylgalactosamine (GalNAc)–yellow square, d-glucose (Glc)–blue circle, *N*-acetylglucosamine (GlcNAc)–blue square, l-fucose (Fuc)–red triangle.

ABO(H) histo-blood group antigens are carbohydrates expressed on the surface of red blood cells and epithelial cells, as glycolipids or glycoproteins. The smallest determinant is the H determinant, a trisaccharide characteristic of blood group O. It can be converted into the A- and B-determinants by enzymatic addition of *N*-acetylgalactosamine (GalNAc) or galactose (Gal), respectively (see Fig 1 for carbohydrate structures and nomenclature). Blood group antigens are also expressed in bodily secretions such as mucus or as free lactose-derived oligosaccharides in human milk (referred to as ‘human milk oligosaccharides’ or HMOs, to distinguish these from ‘blood group antigens’ or BGAs) [19–21].

Despite the identification of the cholera toxin as the likely culprit of cholera blood group dependence [7], the literature has been inconclusive regarding the toxin’s capacity to bind blood group antigens. While the cholera toxin was reported to bind A- and B-active glycolipids and glycoproteins on intestinal brush border membranes [22, 23], microtiter well assays suggested that only a chimera of CTB and the homologous heat-labile enterotoxin from enterotoxigenic *E*. *coli* (LTB), but not CTB itself, could bind blood group antigens [24]. Two crystal structures of LTB and the CTB/LTB chimera in complex with HMOs identified a possible blood group antigen binding site on the lateral side of the toxins, distinct from its primary binding site (for GM1) [25, 26]. Recent studies, by surface plasmon resonance (SPR), isothermal titration calorimetry (ITC) and saturation transfer difference NMR (STD NMR), confirmed that HMOs or other blood group antigen derivatives can indeed bind to CTB, however, none of these studies used unmodified blood group determinants [27–29]. Furthermore, they came to different conclusions: While HMOs were found to bind to CTB of both biotypes, but with different kinetics [27], a custom-synthesized β-glycoside of the B Lewis-y oligosaccharide only bound to cCTB, but not to ET CTB [28]. The binding to ET CTB was restored by mutating Ile47 to Thr47, suggesting a prominent role of this residue in blood group antigen recognition.

The current study was designed to elucidate the molecular causes of cholera blood group dependence. Here, we present high-resolution X-ray crystal structures of both variants of CTB, in complexes with the unmodified blood group determinants of blood groups A and O, with matching SPR data.

## Results

### Crystal structures

#### Blood group determinants bind to the lateral side of the cholera toxin

**Fig 2:**
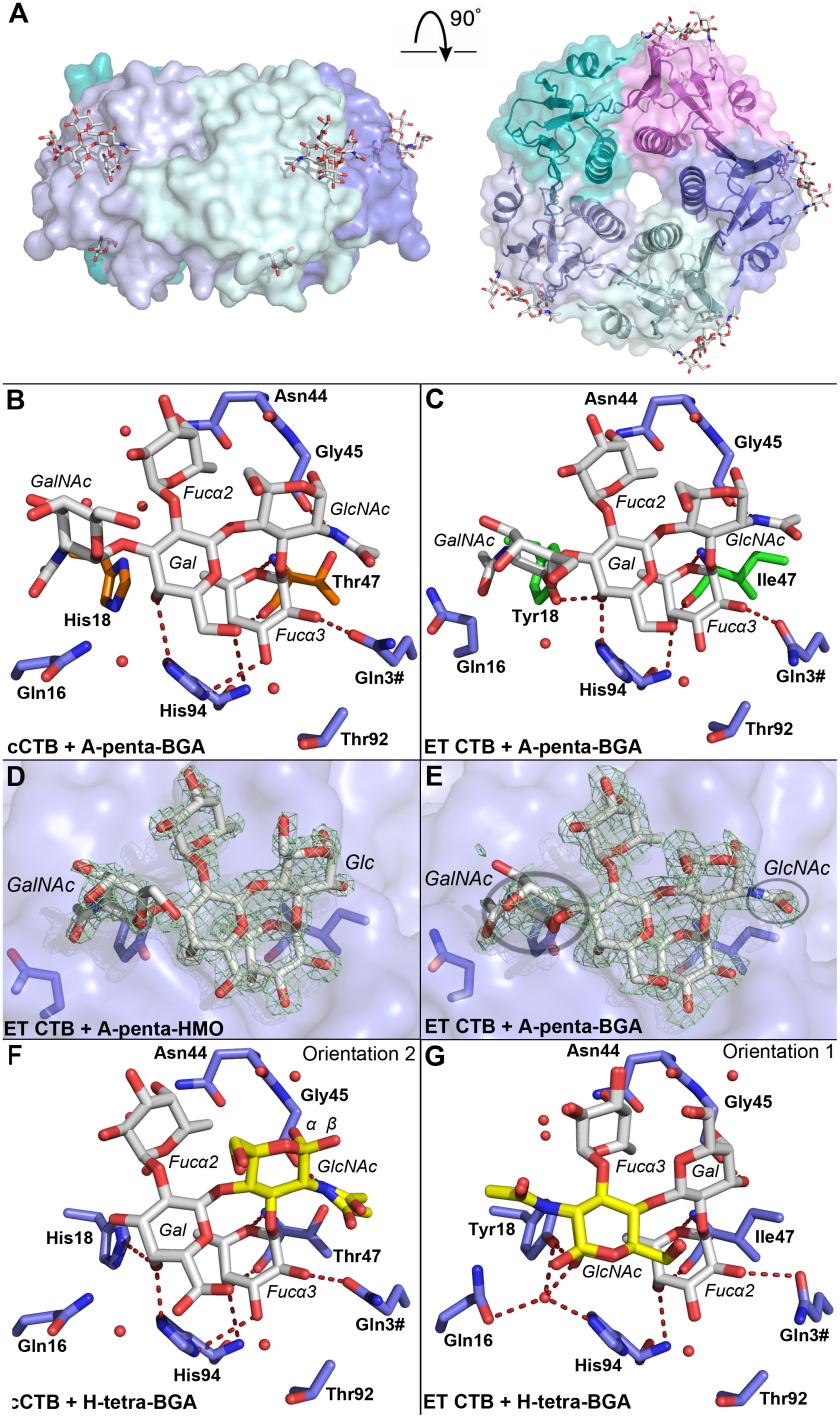
Blood group antigen binding to the cholera toxin. (**A**) X-ray structure of cCTB in complex with A-penta-BGA (PDB ID: 5ELD); side and top views. The B-pentamer is colored by subunit, and the ligands are shown in stick representation. Blood group determinants are bound to the lateral side of the toxin (modeled into four of the five secondary binding sites), and galactose to the primary binding site (in three of the sites), facing the cell membrane. (**B-G**) Close-up views of the blood group antigen binding sites. Oligosaccharide residues are labeled in italics, amino acid residues in bold, and residues from neighboring subunits are indicated with a hash (#). (**B-C**) Interactions of the blood group A determinant with cCTB and ET CTB. Biotype-specific residues are highlighted in orange and green sticks. Water molecules are shown as red spheres, and hydrogen bonds are depicted as red dashed lines. (**D-E**) Electron density. *σ*_A_-weighted *F*_o_ − *F*_c_ maps (green mesh; contoured at 3.0*σ*) are shown for the blood group A determinant and a human milk oligosaccharide. The maps were generated before insertion of the ligands. Circles indicate special features: the GlcNAc’s *N*-acetyl group, and the less-defined electron density for GalNAc in A-penta-BGA. (**F-G**) Interaction of the blood group H determinant with cCTB and ET CTB (two orientations). Yellow residues mark the reducing-end GlcNAc, α and β anomers are labeled. Water molecules are depicted as red spheres, hydrogen bonds represented by red dashed lines. In B, C, F and G, only those H-bonds are shown that have favorable angles, maximum bond lengths of 3.5 Å and that are conserved in all binding sites.

Altogether, five X-ray crystal structures were determined in this study, revealing for the first time the atomic details of blood group antigens bound to the cholera toxin (Fig 2). Four of the structures contain the blood group determinants A-penta-BGA and H-tetra-BGA (bound to cCTB and ET CTB, respectively), while the fifth structure is in complex with the human milk oligosaccharide A-penta-HMO (bound to ET CTB). The structures were solved to 1.1–1.6 Å resolution and refined to *R*_free_ values of 15.6–26.0% (Table 1). These are the first published cholera toxin structures of the El Tor biotype. Moreover, no previous toxin structure has been reported in complex with the blood group H determinant, which is characteristic of blood group O.

**Table 1:** Data collection and refinement statistics.

Data set	H-tetra-BGA		A-penta-BGA		A-penta-HMO
CTB biotype	cCTB	ET CTB	cCTB	ET CTB	ET CTB
ESRF Beam line	ID29	BM30A1	ID29	ID29	ID29
Space group	*P*2_1_2_1_2_1_	*P*2_1_(twinned)	*P*2_1_2_1_2_1_	*P*2_1_2_1_2_1_	*P*2_1_2_1_2_1_
Unit cell parameters					
a (Å)	72.3	63.7	69.7	63.5	63.6
b (Å)	99.0	80.9	69.8	83.0	84.7
c (Å)	152.0	95.7	134.7	196.5	195.7
β (°)	90	96.1	90	90	90
Resolution (Å)	49.5–1.1	47.6–1.5	48.5–1.4	49.1–1.6	48.9–1.6
	(1.14–1.08)	(1.59–1.50)	(1.48–1.40)	(1.70–1.60)	(1.64–1.55)
No. of unique reflections	369957	146071	129054	136523	153547
	(14508)	(21274)	(20478)	(21669)	(24396)
Redundancy	5.7 (2.7)	2.6 (2.4)	4.4(4.2)	4.7 (4.5)	5.2 (5.0)
Completeness (%)	78.9 (19.3)	94.4 (85.2)	99.1 (98.1)	99.2 (98.6)	99.6 (98.8)
*I/σ* (*I*)	12.5 (1.0)	9.2 (1.3)	10.9 (1.3)	7.8 (0.9)	9.2 (1.2)
*R*_meas_	7.0 (101)	8.0 (79)	7.6 (113)	12.6 (116)	12.7(125)
CC_1/2_	99.9 (52.0)	99.7 (55.0)	99.8 (46.7)	99.7 (40.4)	99.8 (41.6)
*R*_cryst_ / *R*_free_ (%)	12.1 / 15.6	22.1 / 26.0	17.1 / 19.4	18.3 / 22.7	17.9 / 22.0
*r*.*m*.*s*.*d*. bond lengths (Å)	0.021	0.015	0.015	0.014	0.017
*r*.*m*.*s*.*d*. bond angles (°)	2.22	1.96	1.81	2.11	1.90
Average *B*-factor (Å^2^)					
Backbone	11.6	17.8	15.0	19.3	18.0
Side chains + solutes	18.6	21.1	19.9	24.0	23.2
Oligosaccharides	18.7	25.3	22.8	31.1	30.2
All atoms	16.0	19.7	18.0	22.0	21.1
Ramachandran plot profile (%)					
Favored	97.4	97.6	97.6	97.3	97.4
Allowed	2.6	2.4	2.4	2.7	2.6
Disallowed	0.0	0.0	0.0	0.0	0.0
PDB ID	5ELB	5ELC	5ELD	5ELE	5ELF

Values in parentheses refer to the highest resolution shells.

All structures exhibit the characteristic fold of the cholera toxin B-pentamer [10, 30] (Fig 2A). The crystals grown in Tris-bicine buffer (the ET CTB complexes and cCTB + H-tetra-BGA) contained two B-pentamers in the asymmetric unit (arranged “top-to-top”; S1 Fig), while the cCTB + A-penta-BGA crystals, grown in MES-imidazole buffer, contained only a single B-pentamer. In all five structures, the blood group oligosaccharides were found to bind to the lateral side of the toxins, between the biotype-specific residues 18 and 47. The same site was previously identified in crystal structures of LTB and a CTB/LTB chimera, in complexes with A-penta-HMO [25, 26]. Except for the loop region comprising residues 50–61 (in some of the subunits) and the C-terminal residue Asn103, the electron density is well defined in all structures. In addition to the ligands at the secondary binding site, the two structures of cCTB contain Gal monosaccharides in the primary binding site (Fig 2A), which shows that the toxin can bind ligands at both binding sites simultaneously. Galactose is presumably a remnant from the purification by D-Gal affinity chromatography.

#### A-penta-BGA binds to CTB in an unexpected orientation

Crystal structures of the cCTB and ET CTB complexes with A-penta-BGA were determined to a resolution of 1.4 Å and 1.6 Å, respectively. The crystals were obtained from different crystallization conditions and were non-isomorphous, but belonged to the same crystallographic space group (*P*2_1_2_1_2_1_, Table 1). In both structures, A-penta-BGA is bound with the blood-group-A-specific GalNAc residue facing amino acid 18, and with its reducing end (*N*-acetylglucosamine; GlcNAc) towards residue 47 (Fig 2B and 2C). This is in stark contrast to the previously published structures of LTB and the CTB/LTB chimera, where A-penta-HMO is bound in the opposite orientation, with the reducing-end glucose (Glc) facing towards Tyr18 [25, 26] (referred to here as orientation 1; S2 Fig). The only sequence differences in the binding sites of the CTB/LTB chimera and the ET CTB structures concern residues 47 and 94, represented by Thr and Asn in the chimera, and by Ile and His in ET CTB, respectively. In the ET CTB structure, only three of the ten binding sites exhibited sufficient electron density to allow modeling of the entire ligand. Even in those cases, the *B*-factors of the terminal carbohydrate residues GalNAc and Fucα2 were disproportionally high compared to those in the other structures, suggesting that they are less tightly bound (Fig 2E; in cCTB, the electron density of the GalNAc residue is slightly better defined). Moreover, Fucα2 displays only water-mediated and internal carbohydrate-carbohydrate interactions, whereas the trisaccharide core (Galβ4[Fucα3]GlcNAc) consistently shows strong H-bonding interactions with CTB (Fig 2B and 2C, S1 Table). Fucα3 is particularly tightly anchored to the toxin in both structures, through hydrogen bonds with the backbones of residues 47 and 94 as well as the side chain of Gln3 from the neighboring B-subunit. The reducing-end GlcNAc binds to the backbone of Gly45. The main differences between the cCTB and ET CTB structures concern the internal Gal residue, which interacts with Tyr18 and His94 in the ET CTB structure, while in cCTB, it is too far away from the shorter His18 side chain to form a hydrogen bond (Fig 2B and 2C). It is noteworthy that in the ET CTB structure exclusively, Gln16 often has a different conformation and points away from the ligand-binding site, thereby weakening the H-bonding network to the ligand.

#### A-penta-HMO binds to CTB in the same orientation as A-penta-BGA

To compare the results from the CTB structure in complex with A-penta-BGA with those obtained with the related human milk oligosaccharide used in previous investigations [25–27, 29], A-penta-HMO was co-crystallized with ET CTB, and the structure was refined to a resolution of 1.6 Å. The ligand binds in the same orientation as A-penta-BGA in the other CTB structures (here referred to as orientation 2). The complete ligand could be reliably modeled in seven of ten binding sites, with the terminal GalNAc and Fucα2 residues being far more ordered than in A-penta-BGA (Fig 2D and 2E). In both structures, Gln16 points away from the ligand in the highly occupied sites. Otherwise, Gln16 adopts the original conformation.

#### H-tetra-BGA binds to CTB in two orientations

To our knowledge, this is the first structure of an AB_5_ toxin (and in fact of any protein toxin) in complex with the blood group H determinant. The crystal structures of the cCTB and ET CTB complexes were determined to a resolution of 1.1 Å and 1.5 Å, respectively. Both proteins crystallized using the same conditions, but in different space groups (*P*2_1_2_1_2_1_ and *P*2_1_; Table 1). Well-defined electron density is present in all ten binding sites of the two pentamers, although the ligand orientations differ. While the electron density of the cCTB structure primarily supports orientation 2, most ET CTB binding sites exhibit the ligand in orientation 1 (Fig 2F and 2G). Since H-tetra-BGA is an almost symmetrical molecule, the fucosyl residues are indistinguishable. As for the complexes with A-penta-BGA, the fucose near residue 47 is most tightly anchored to the toxin, while the other fucose is loosely bound, by indirect water-mediated interactions. In general, the two structures are very similar, except that the *N*-acetyl group changes place in the two different orientations; the axial 4-OH group of Gal is mimicked by the α-anomer of GlcNAc (Fig 2F, 2G and S3 Fig). In orientation 1, the *N*-acetyl group takes the position of the GalNAc residue characterizing A-penta-BGA. This again points to the Galβ4[Fucα3]GlcNAc trisaccharide being the central binding motif, with additional terminal residues being less important or possibly even disruptive for binding.

### Surface plasmon resonance

**Fig 3:**
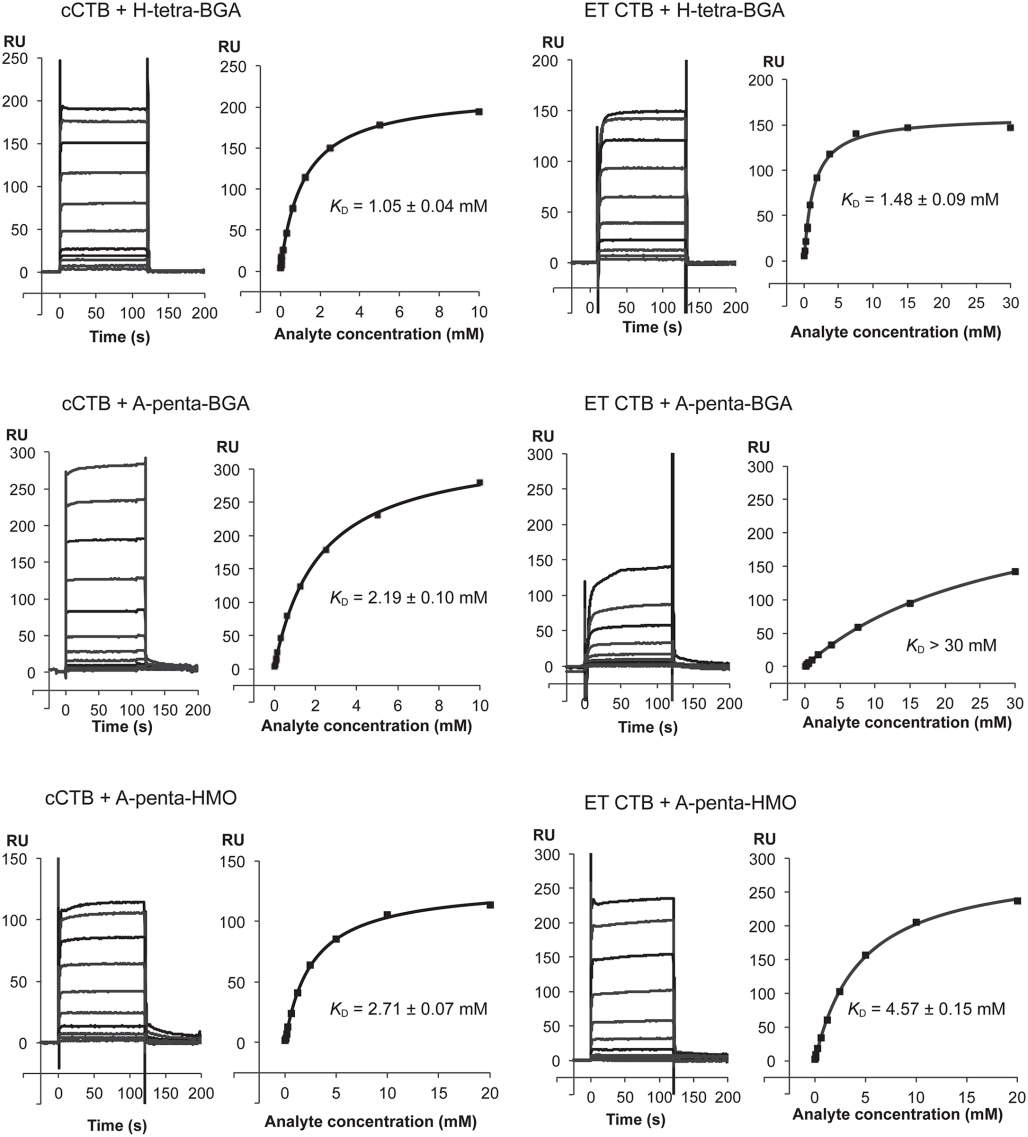
Representative SPR sensorgrams and affinity plots. SPR experiments were performed in triplicates, with cCTB or ET CTB coupled to the sensor chip and using blood group determinants or A-penta-HMO as analytes, as indicated in the panel legends. The final *K*_D_ values are shown with standard deviations. The *K*_D_ value for ET CTB + A-penta-BGA could not be calculated since it did not reach saturation upon addition of 30 mM ligand. The experiments with A-penta-HMO were done at a separate occasion; therefore the RU-axes are not comparable. Details are provided in the Materials and Methods section.

Binding of the different blood group oligosaccharides to cCTB and ET CTB was further characterized by SPR (Fig 3). CTB was immobilized on the sensor chip, and the oligosaccharides were injected in increasing concentrations. H-tetra-BGA was found to bind both biotypes equally well (*K*_D_ = 1.1/1.5 mM for cCTB/ET CTB), whereas A-penta-BGA bound cCTB significantly stronger than ET CTB (*K*_D_ = 2.2/> 30 mM for cCTB/ET CTB). In contrast, A-penta-HMO exhibited similar binding affinities (*K*_D_ = 2.7/4.6 mM for cCTB/ET CTB), comparable to previously reported values (*K*_D_ = 1.2 mM, as determined by SPR for both cCTB and ET CTB [27]; *K*_D_ = 5.2/6.4 mM for cCTB/ET CTB, as determined by STD-NMR [29]). However, the new experiments showed no differences in the kinetics; all ligands exhibited the same quick association and dissociation, and no regeneration of the SPR chips was required. Preliminary experiments with B-penta-BGA (Elicityl SA) were also performed and showed the same phenomenon as for A-penta-BGA (*K*_D_ > 30 mM), suggesting a strong effect of the *N*-acetyl group present in blood group antigens. In addition, we performed preliminary SPR experiments with a blood group A determinant of type-1 structure, which showed very weak to no binding to CTB of both biotypes. Blood group antigens with type 2 core structures are well represented on small intestinal glycoproteins [31], while type 1 structures are predominantly found on glycolipids [32]. We conclude that the toxins prefer type 2 over type 1 structures, in line with previous experiments [24, 33].

## Discussion

We solved the X-ray crystal structures of cCTB and ET CTB in complexes with the blood group A and H determinants, with the ambition to reveal the molecular origins of cholera blood group dependence, and followed this work up with quantitative binding analysis. We chose to work with A rather than B determinants, since A-determinants differ the most from the H-determinants. This choice also facilitates comparison with previous crystal structures. We here present the first published X-ray structures of any naturally occurring CTB variant in complex with blood group determinants, the first structures of ET CTB, and the first toxin structures in complex with the blood group H determinant. In addition we could show that the primary and secondary binding sites of the toxin can be occupied simultaneously (Fig 2A).

### ET CTB distinguishes more strongly between A/B- and H-antigens than cCTB

CTB of both *V*. *cholerae* biotypes were found to bind blood group determinants or HMOs at a secondary binding site on the lateral face of the toxin (Fig 2A). The same site was identified in previous studies for A-penta-HMO binding to LTB or a CTB/LTB chimera [25, 26]. However, the oligosaccharides exhibited unexpected orientations: both A-penta-BGA and A-penta-HMO bound to CTB in the opposite orientation compared to the other toxin structures, and H-tetra-BGA bound in both orientations. Complementary SPR analysis revealed that while both cCTB and ET CTB bound to H-tetra-BGA with similar affinity (1.0 and 1.5 mM, respectively, which is comparable to H-tetra-HMO, *K*_D_ = 0.5 mM for ET CTB [27]), they differed greatly in their binding affinity to A-penta-BGA (with ET CTB binding very weakly). Similar observations were previously made using ITC, involving a β-glycoside of B-penta-BGA [28]. The millimolar *K*_D_ values observed for the ABH blood group oligosaccharides may seem modest, but multivalent binding of the pentamer is expected to enhance binding avidity significantly. This has been demonstrated for GM1 [34], and also for Shiga toxins, which bind the GD3 ganglioside with millimolar affinity, but achieve nanomolar avidity upon multivalent binding [35]. Summarizing, our experiments revealed that ET CTB can strongly distinguish between blood group A and H determinants, which both contain a GlcNAc residue at the reducing end, where human milk oligosaccharides contain unmodified Glc. In the ET CTB crystal structure, only three of ten binding sites showed sufficient electron density to warrant modeling of the entire A-penta-BGA ligand, and even then, the GalNAc residue characteristic of blood group A was disordered (Fig 2E). Notably, the GalNAc residue is close to Tyr18 and not Ile47, the substitution of which restored binding activity [28]. This was in contrast to expectations, since the ligand bound in the opposite orientation compared to the toxin structures published previously [25, 26]. In the crystal structure of ET CTB in complex with A-penta-HMO, the GalNAc residue is, however, well defined by electron density (Fig 2D), strongly suggesting that interference of the reducing end *N*-acetyl group with Ile47 is responsible for the weaker binding affinity of A-penta-BGA *versus* A-penta-HMO and ET CTB *versus* ET CTB I47T/cCTB/LTB. In addition, we observe a conformational change of Gln16 upon binding of A-penta (BGA or HMO), which suggests that this residue interferes with binding in its native conformation.

### The structures likely represent a biologically relevant scenario

The trend appears clear: The cholera toxin binds blood group H-determinants preferentially over A-determinants, suggesting that stronger binding correlates with more severe disease. The relationship is especially pronounced for the El Tor biotype, which shows the strongest blood group dependence [17]. Individuals with the ‘secretor’ phenotype, who also express ABO(H) blood group antigens on the surface of intestinal epithelial cells and mucins, experience less severe symptoms than ‘non-secretors’ [36]. How can this be explained? The small intestinal mucus layer is known to serve a protective function, by blocking and removing intruding pathogens and toxins. Stronger binding may hence be expected to correlate with enhanced protection against disease rather than with increased toxicity. However, binding to the mucus layer can serve as a double-edged sword: by binding potent invaders, the invaders can also gain entrance to the host cells. Moreover, with the toxins likely being released in close proximity to the host cells [37, 38], the main barrier may already be overcome.

But are the measured affinities at all biologically relevant? For one, only the H-determinant, but not the A-determinant, can bind to the toxin in two alternative orientations, likely contributing to its increased affinity. Both orientations should in principle be possible in the context of glycoconjugates, as long as they are β-glycosidically linked, avoiding clashes with the toxin. In this regard, we note that there is a dominance of the α-anomer of the reducing end GlcNAc in the X-ray structures, mimicking the Gal 4-OH group, while the relevant glycoconjugates are β-glycosidically linked (S2 Fig). This may influence binding affinities, and the actual differences between blood group determinants with fixed β-linkage may be even greater. Also, we need to consider that the blood group determinants are terminal epitopes of glycoconjugates, and are connected to the protein/lipid by carbohydrate linkers. The lengths of these linkers can also have an effect on the binding to CT, with shorter linkers preventing the non-reducing end of the glycoconjugates from reaching to blood group binding site of the toxin. Finally, not all blood group antigens exhibit the second fucose residue (Fucα3), which is likely critical for binding in orientation 2. It is very difficult to predict glycosylation based on the expression profiles of glycosyltransferases, and only limited data exist regarding the distribution of glycoconjugates in human tissues [19, 39]. However, it is known that secretor fluids are characterized by enhanced fucosylation [40, 41], hence secretors should be able to bind a considerable fraction of the blood group antigens in orientation 2. In addition, it has recently been shown that fucosylated structures promote CTB binding and cellular uptake in colonic epithelial cells [42]. Therefore we expect that the discrimination between blood group determinants observed *in vitro* resembles the situation in the human body.

### Model of the molecular basis of cholera blood group dependence

**Fig 4:**
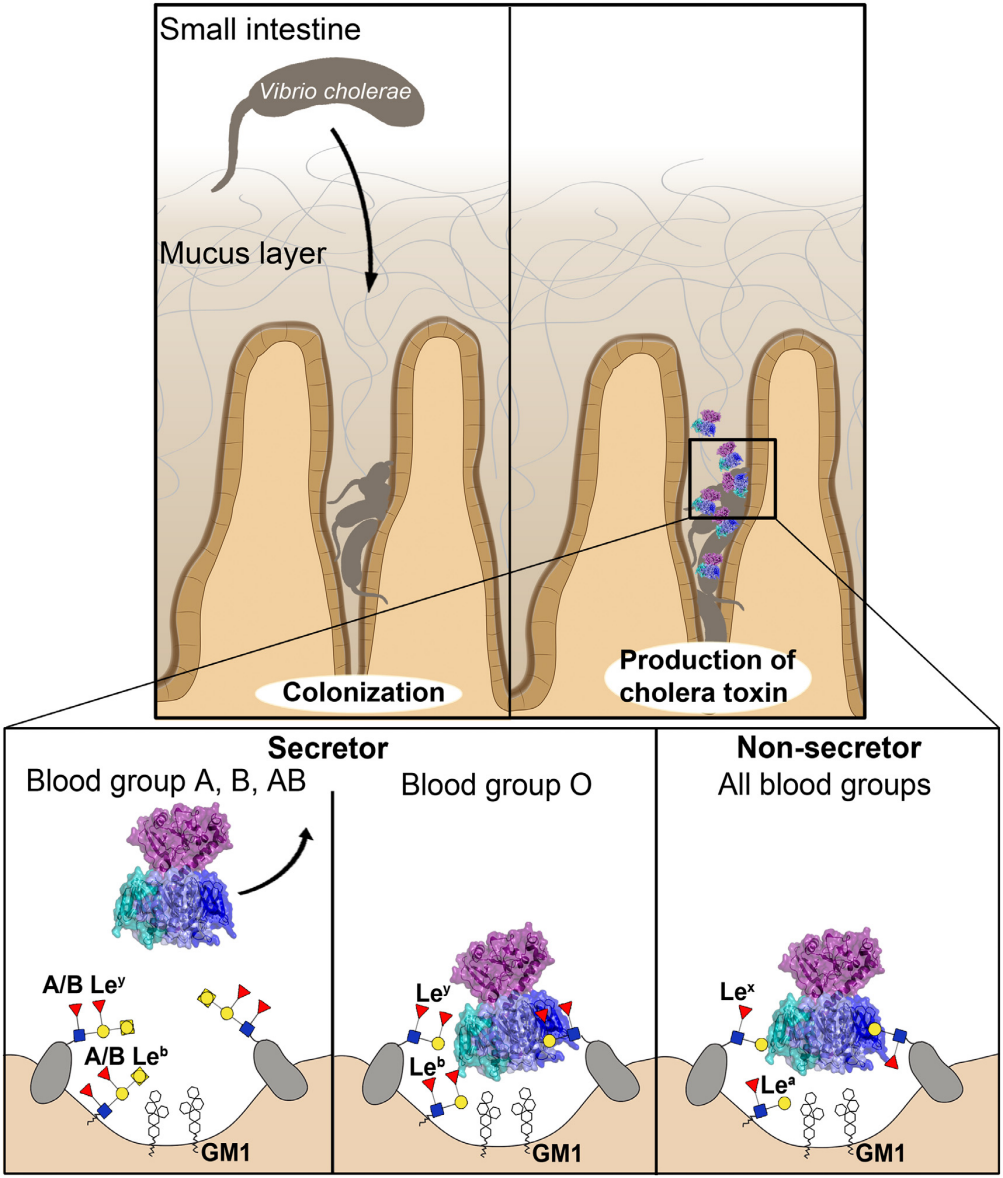
Molecular model of cholera blood group dependence. *V*. *cholerae* burrows through the mucus layer, aided by its flagellum and mucinases. After colonization, it produces the cholera toxin. CT’s interaction with the host cells depends on the person’s blood group and secretor status. In secretors, blood group antigens are expressed on mucins and intestinal epithelial cells. H-antigens (characteristic of blood group O) can bind the toxin in two orientations. They bind the toxin more strongly than A-antigens and therefore retain the toxin, increasing the risk of cellular uptake. CT has a lower affinity to A-antigens, and is therefore more easily ejected by the peristaltic movement of the intestine. This protects secretors with blood group A from severe disease, while blood group O individuals suffer more serious symptoms.

The scenario may be as follows (Fig 4): After colonization of *V*. *cholerae* in the human small intestine, the bacteria secrete their main virulence factor, the cholera toxin. The small intestine is well suited for invasion, because of the limited thickness of its mucus layer, as compared to, for example, the colon. At the Peyer’s patches, the mucus layer is particularly thin. Moreover, after efficient intestinal colonization, the bacteria are already in close contact with the epithelium [37, 38]. The secreted toxin either binds directly to the receptors on the epithelial cells or to glycoconjugates in the human mucus layer—among those, if present (in secretors), the ABH blood group antigens. Also non-secretors are likely to exhibit suitable docking structures (such as Lewis-x (Galβ4[Fucα3]GlcNAcβ), which comprises part of H-tetra-BGA), but only secretors contain ABH histo-blood group antigens in their mucus and on intestinal epithelial cells [19, 39]. Low-affinity multivalent receptor interactions with quick association-dissociation dynamics are likely essential for efficient intoxication, to ensure that the toxin reaches the epithelial cells and does not get stuck on its way (and risk being expelled by mucus shedding). Once the CT reaches the epithelial cells, it may either bind to the GM1 ganglioside, to other functional receptors [43–45], or be engulfed by an alternative mechanism involving the turn-over of brush border membranes [46–48]. Here, it is interesting to note that based on the data presented here, the CT appears to be able to bind to blood group antigens and GM1 simultaneously. However, until the toxin is strongly bound to the cell surface, it may still diffuse away from the cells through the mucus layer. The flow around the small intestinal villi is poorly understood, but simulations suggest that during intestinal contractions, mixing and absorption around the mucosa is increased [49]. Diffusion of the toxin away from the epithelium towards the lumen of the gut is more likely to occur in secretors with blood group A or B than in those with blood group O, which bind the CT more strongly, via their H-antigens. The toxin is therefore expected to be able to enter host cells more effectively in secretors with blood group O than in those with blood groups A or B, with the effect being most pronounced for the El Tor biotype. How this compares to non-secretors is unknown, but it is conceivable that the steric interference observed for blood group A and B determinants is the main protecting factor from severe disease.

Such a scenario would explain why secretors with blood groups A, B or AB are relatively protected, while those with blood group O experience more severe symptoms, as observed by Arifuzzaman *et al*. [50]. With ≈80% of the human population being secretor-positive, individuals with blood group O would overall be more prone to severe disease, as observed [7, 8, 17]. Likewise, they would experience stronger protection from vaccination [17]. The scenario further explains why the El Tor biotype is more discriminatory than the classical biotype [17]. However, one should keep in mind that in recent years, a new hybrid biotype evolved, which expresses a toxin with the classical sequence [18]. This enables more effective intoxication also of individuals with blood group A or B, promoting the bacteria’s propagation.

### Presence of GM1 could explain conflicting SPR data

With respect to the human milk oligosaccharides, we note that previous SPR data [27] showed different binding kinetics for H-tetra-HMO and A-penta-HMO, with A-penta-HMO exhibiting significantly slower association/dissociation rates. In contrast, all ligands used in the present study showed fast binding and release. The ligands used were obtained from different sources, but were otherwise identical. While the previous study was performed with compounds isolated from the feces of breast-fed infants (Isosep AB), the SPR results presented herein are based on ligands produced by bacterial fermentation (Elicityl SA). In both cases, A-penta-HMO samples were only > 90% pure, suggesting that residual impurities may have contributed to the different binding profiles. Indeed, the SPR binding profile in our earlier report (Fig 1CE in [27]) suggests two separate binding events upon closer inspection. GM1 and GM1-derived oligosaccharides have earlier been shown to be present in human milk [51, 52], and are known to strongly bind CTB [13, 53]. The presence of trace amounts of these would explain the observed binding profiles. Preliminary ELISA experiments (S4 Fig and S1 Methods) support such an interpretation.

### Conclusions and perspective

While cell biological studies are needed to establish the molecular basis of cholera blood group association, the picture becomes increasingly clear. The additional GalNAc (or Gal) residue in blood group A (or B) antigens weakens binding to the CT through steric interference. Moreover, only the symmetric blood group H (but not A) determinants can bind to the CT in two alternative orientations. This enhances the binding capacity and the biological potency of H-antigens. While in endemic regions like Bangladesh, the blood group profile of the population has been shaped by evolution to minimize losses of human lives [3, 29, 54], most of the world is less well adapted. With climate change, water-borne diseases like cholera are predicted to be aggravated worldwide [55, 56], with significant socioeconomic costs [57]. The molecular insights obtained in this investigation may help to stem the tide.

## Materials and Methods

### Cloning of the cCTB gene

The nucleotide sequence of the classical CTB gene (Genbank: AAC34728.1; coding for amino acid sequence 22–123) was codon-optimized for expression in *Escherichia coli* and synthesized by GeneArt (Life Technologies) in a standard pMA-T plasmid. The gene contained the N-terminal LTB signal sequence directing the protein to the periplasmic space, as well as flanking *Nde*I and *Bam*HI restriction sites. After excision of the gene, it was ligated into a pET-21b(+) vector (Novagen), and transformed by heat shock into competent *E*. *coli* BL21 (DE3) cells for protein expression.

### Expression and purification of cCTB

The cells transformed with the pET-21b(+)-cCTB plasmid were grown in LB medium supplemented with 0.1 mg/ml ampicillin at 37°C until an OD_600nm_ of ~0.5 was reached. Before induction with 0.5 mM IPTG, the temperature was lowered to 25°C and the protein was expressed for 16–20 hours. The cells were harvested by centrifugation and re-suspended in ice-cold sucrose solution (20 mM Tris pH 8, 25% (w/v) sucrose, 5 mM EDTA), and incubated on ice for 15 minutes. The supernatant was removed by centrifugation at 8500 × g for 20 minutes, and the pellet was re-suspended in periplasmic extraction buffer (5 mM MgCl_2_, 0.1 mg/ml lysozyme). The periplasmic fraction was separated from the cell debris by centrifugation at 8500 × g for 20 minutes, and dialyzed against PBS. cCTB was purified by affinity chromatography using D-Gal-sepharose (Thermo Scientific), and was eluted with 300 mM D-Gal (AppliChem) in PBS. The protein was concentrated and applied to a Superdex75 size-exclusion chromatography column (GE Healthcare), where the buffer was exchanged to 20 mM Tris pH 7.5, 100 mM NaCl. The purified protein was dialyzed against Tris-storage buffer (20 mM Tris pH 7.5, 200 mM NaCl), concentrated to 3–10 mg/ml and stored at -80°C.

### Expression and purification of ET CTB

*Vibrio* sp. 60 containing the gene for ET CTB was grown in LBS medium (LB medium with 15 g/l NaCl) at 30°C, supplemented with 0.1 mg/ml ampicillin, until an OD_600nm_ of ≈0.2. The culture was induced with 0.5 mM IPTG and expressed for 16–20 hours. ET CTB is naturally secreted into the growth medium, and the cells were removed by centrifugation at 40,000 × *g*. The supernatant was applied to a D-Gal-sepharose affinity column, and ET CTB was eluted using 300 mM D-Gal in PBS. After concentration, the protein was further purified by size-exclusion chromatography (in 20 mM Tris pH 7.5, 100 mM NaCl) and dialyzed against 20 mM Tris pH 7.5, 200 mM NaCl. The purified protein was concentrated to 3–10 mg/ml, and stored at -80°C.

### Crystallization

CTB and oligosaccharide ligands were mixed at a molar ratio of 1:10 (B-subunit:ligand) two hours prior to the crystallization setups. For the initial screening performed on an Oryx4 crystallization robot (Douglas Instruments, UK), the sitting-drop vapor-diffusion technique was used, with a CTB concentration ranging from 3.0 to 8.5 mg/ml at 20°C. For the optimization setups, the hanging-drop vapor-diffusion technique was used with the same protein concentration range, also at 20°C. All oligosaccharides were purchased from Elicityl-Oligotech (Elicityl SA, Crolles, France), with the product numbers GLY035-5 (A-penta-BGA: GalNAcα3[Fucα2]Galβ4[Fucα3]GlcNAc), GLY048 (H-tetra-BGA: Fucα2Galβ4[Fucα3]GlcNAc) and GLY067 (A-penta-HMO: GalNAcα3[Fucα2]Galβ4[Fucα3]Glc). Crystals of the ET CTB complexes were grown in 0.1 M Tris-bicine pH 8.5, 9–12.5% (each) PEG1000/PEG3350/MPD, 30 mM (each) CaCl_2_/MgCl_2_ (optimization of hits from the Morpheus crystallization screen; Molecular dimensions). Crystals of the cCTB complexes were obtained from either 0.1 M Tris-bicine pH 8.5, 9–12.5% (each) PEG1000/PEG3350/MPD, 30 mM (each) CaCl_2_/MgCl_2_, or 0.1 M MES-imidazole pH 6.7, 25% PEG4000, 5% PGA-LM (optimization of hits from the PGA-LM crystallization screen; Molecular Dimensions). Diffraction quality crystals were obtained by microseeding, using microseed stocks prepared by crushing small crystals from initial hits with a seed bead. Most of the CTB complexes crystallized in space group *P*2_1_2_1_2_1_, with two pentamers in the asymmetric unit, from a MORPHEUS-derived condition, except for ET CTB + H-tetra-BGA, which crystallized in *P*2_1_, and cCTB + A-penta-BGA, which crystallized from the PGA-LM-derived condition, with one pentamer in the asymmetric unit (space group *P*2_1_2_1_2_1_). Unit cell parameters are listed in Table 1.

### Data collection and refinement

Diffraction data for all complexes were collected at ESRF beam lines ID29 [58] or BM30A-1 (automated data collection by MASSIF-1). Data collection and refinement statistics are listed in Table 1. Data were processed and scaled using XDS [59] and Aimless from the CCP4 software suite [60], and the structures solved by molecular replacement using MOLREP [61] or Phaser [62], taking the atomic coordinates of the 1.25 Å crystal structure of CTB as a search model for molecular replacement (PDB: 3CHB [63]). The search model was prepared with the program CHAINSAW [64], removing ligands, water molecules and alternative conformations, as well as pruning non-conserved residues down to the γ atom. After molecular replacement, rigid body refinement was performed with REFMAC5 [65], using 5% of the data for cross-validation (*R*_free_). The structures were subsequently manually optimized using Coot [66] and further refined with REFMAC5. Automated restrained refinement and manual rebuilding were repeated in an iterative manner until no more significant changes in *R*-factors were observed. The ET CTB + H-tetra-BGA structure exhibited twinning (twin fraction 0.1), and was consequently refined using intensity-based twin refinement as implemented in REFMAC5. The highest-resolution structure, cCTB + H-tetra-BGA (1.08 Å), was refined with anisotropic *B*-factors. In all structures, the sequence was corrected by substituting His94 for Thr94, and in the ET CTB structures, in addition His18 and Thr47 were replaced by Tyr18 and Ile47, respectively. After refinement of the protein alone, ordered buffer components and ions were modeled, before adding water molecules in several cycles. The blood group or human milk oligosaccharides were included last, to ensure unbiased and conformationally optimal modeling. The ligands were prepared with MarvinSketch (ChemAxon.com), using isomeric SMILES strings for the individual monosaccharides. The final ligand PDB and library files were created using PRODRG [67], and were checked for errors using pdb-care (glycosciences.de/tools/pdbcare [68]). The oligosaccharides were only modeled into the binding sites if clear electron density of at least three residues was present. Occupancies were refined by comparing the *B*-factors of the ligands with those of the interacting protein atoms, and taking the difference Fourier maps into account. The structures were validated using Coot [66], Molprobity [69], and figures prepared using PyMol (Schrödinger LLC).

### Surface plasmon resonance

SPR analyses were performed on a Biacore T100 biosensor system (Biacore Life Sciences, GE Healthcare, Uppsala, Sweden) at the Infrastructural Centre for Analysis of Molecular Interactions, University of Ljubljana, Slovenia. The interaction studies were carried out at 25°C in HBS-EP running buffer (10 mM Hepes pH 7.4, 150 mM NaCl, 3 mM EDTA, 0.05% (v/v) surfactant P20); and the carbohydrate ligands A-penta-BGA, H-tetra-BGA, A-penta-HMO were solubilized in the same buffer. ET CTB and cCTB were diluted in 10 mM Na-acetate pH 5.5 and immobilized by amine coupling to a CM5 sensor chip to a response of 4000–7000 RU. For the first experiments with cCTB, H-tetra-BGA and A-penta-BGA were injected over the surfaces with an initial flow rate of 20 μl/min for 120 s (followed by a 60 s dissociation phase and allowing 60 s of stabilization prior to the next injection), at increasing concentrations of the carbohydrate ligand (20 μM to 10 mM). The experiment was repeated with two injections per concentration at 10 μl/min for 120 s. H-tetra-BGA and A-penta-BGA (58 μM to 30 mM) were injected over the ET CTB chip with an initial flow rate of 20 μl/min for 120 s. The experiment with H-tetra-BGA was repeated with two injections per concentration (29 μM to 15 mM) at a flow rate of 10 μl/min for 120 s. Finally, both ligands were tested against both toxin biotypes using three randomized injections per concentration (313 μM to 10 mM), at a flow rate of 5 μl/min for 30 s. A-penta-HMO was tested at a separate occasion, using the same chip for cCTB (quality tested by a repetition of H-tetra-BGA), but a newly immobilized chip for ET CTB (≈6000 RU). The graphs for A-penta-HMO are therefore not comparable to the rest. The experiments with A-penta-HMO (39 μM to 20 mM) were performed with a flow rate of 10 μl/min for 120 s, with three separate runs. No regeneration was required for any of the interactions. The dissociation constants (*K*_D_) of the interactions were calculated by using a steady-state affinity model in the Biacore T100 evaluation software. Preliminary experiments were performed with B-penta-BGA (GLY38-5, Galα3[Fucα2]Galβ4[Fucα3]GlcNAc) (Elicityl SA) and A-penta-BGA-type-1 (GLY35-4, GalNAcα3[Fucα2]Galβ3[Fucα4]GlcNAc) (Elicityl SA). The binding of B-penta-BGA was measured with a single concentration series from 39 μM to 20 mM (5 μl/min, 60 s) for cCTB, and from 39 μM to 10 mM (5 μl/min, 60 s) for ET CTB, while A-penta-BGA-type-1 was only manually tested at 1 mM and 5 mM concentrations for cCTB, and at 1 mM for ET CTB.

## Supporting Information

S1 Methods Enzyme-linked immunosorbent assay (ELISA) with human milk oligosaccharides. (DOCX)

S1 Fig Contents of the asymmetric unit in crystals grown in Tris-bicine buffer. **Left panel.** Two cholera toxin B-pentamers positioned “top-to-top” in the asymmetric unit of cCTB in complex with H-tetra-BGA (PDB ID: 5ELB). H-tetra-BGA is depicted as white sticks, bicine as green sticks, calcium ions as yellow spheres and galactose molecules as magenta spheres. Buffer components facilitate the crystal contact. **Right panel.** Close-up view of the crystal contact between the two B-pentamers, with one glutamate residue from each B-pentamer and two bicine molecules (green) coordinating two calcium ions (yellow).
(TIF)

S2 Fig Comparison of ligand binding to a CTB/LTB chimera versus cCTB. **Left panel.** Human milk oligosaccharide A-penta-HMO in complex with CTB/LTB chimera (PDB ID: 3EFX [26]; orientation 1). **Right panel.** Blood group A determinant A-penta-BGA in complex with cCTB (PDB ID: 5ELD; orientation 2). In both panels, the oligosaccharides are represented by white sticks, with the terminal GalNAc residue highlighted in purple. The relevant amino acid residues are shown as blue sticks, water molecules as red spheres, and hydrogen bonds as red dashed lines. Only hydrogen bonds conserved in most binding sites and with favorable angles are shown.
(TIF)

S3 Fig High-resolution stereo image of H-tetra-BGA binding to cCTB. The oligosaccharide ligand is represented by white sticks, with the reducing-end GlcNAc residue highlighted in yellow. The relevant amino acid residues are shown as blue sticks. Water molecules are represented by red spheres, and hydrogen bonds by red dashed lines, with distances given in Ångström (PDB entry: 5ELB). The final *σ*_A_-weighted 2*F*_o_ − *F*_c_ electron density map is represented by a grey mesh and contoured at 1.0*σ*.
(TIF)

S4 Fig ELISA experiments. 100 ng/ml ET CTB was preincubated with 5 mM A-penta-HMO, 5 mM H-tetra-HMO or 5 μM GM1 ganglioside, and added to the GM1-coated wells. After washing, the remaining toxin was measured by absorbance at 405 nm. The y-axis shows percentage binding, with the control (ET CTB incubated with water) defined as 100%. All the ligands were purchased from Isosep AB. The experiments show that incubation of A-penta-HMO purchased from Isosep AB interfered with binding of CTB to GM1, and may hence contain trace amounts of GM1 or GM1 fragments.
(TIF)

S1 Table Carbohydrate-protein interactions. (DOCX)
