# Supplementary material for: High-Resolution Crystal Structures Elucidate the Molecular Basis of Cholera Blood Group Dependence
Source: PLoS Pathog. 2016 Apr 15;12(4):e1005567. doi: 10.1371/journal.ppat.1005567 (PMC4833353; doi:10.1371/journal.ppat.1005567)
Supplement: S1 Methods — (DOCX) [file ppat.1005567.s001.docx]

**S1 METHODS**

**Enzyme-linked immunosorbent assay (ELISA) with human milk oligosaccharides**

ELISA experiments were performed using Polysorp plates (Nunc) for adsorption of hydrophobic molecules. The GM1 ganglioside was diluted in methanol and incubated in the Polysorp wells overnight. The wells were washed with PBS before incubation with blocking buffer (PBS + 2 % BSA). 100 ng/ml ET CTB was pre-incubated with A-penta-HMO (5 mM, Isosep AB, 55/01 Lot 4116-228), H-tetra-HMO (5 mM, Isosep AB, 45/02 Lot 4156-172), GM1 (5 μM, Isosep AB, 65/04) or buffer (control) for 1 hour at 37 °C before addition to the wells. The tray was incubated with gentle shaking at RT for 10-60 minutes to allow binding of CTB to the GM1-coated plate. The bound protein was detected by 60 minutes incubation with primary (mouse anti-CT, Santa Cruz) and secondary antibody (goat anti-mouse, Santa Cruz), with extensive washing with PBS-Tween between each addition. The secondary antibody was conjugated to the enzyme alkaline phosphatase (AP). Bound CTB was measured by the addition of p-nitrophenyl phosphate (pNPP, Sigma-aldrich), measuring the absorbance at 405 nm. The experiment was run with 4 parallels, and repeated once.
