# Supplementary figures and images for: High-Resolution Crystal Structures Elucidate the Molecular Basis of Cholera Blood Group Dependence
Source: PLoS Pathog. 2016 Apr 15;12(4):e1005567. doi: 10.1371/journal.ppat.1005567 (PMC4833353; doi:10.1371/journal.ppat.1005567)

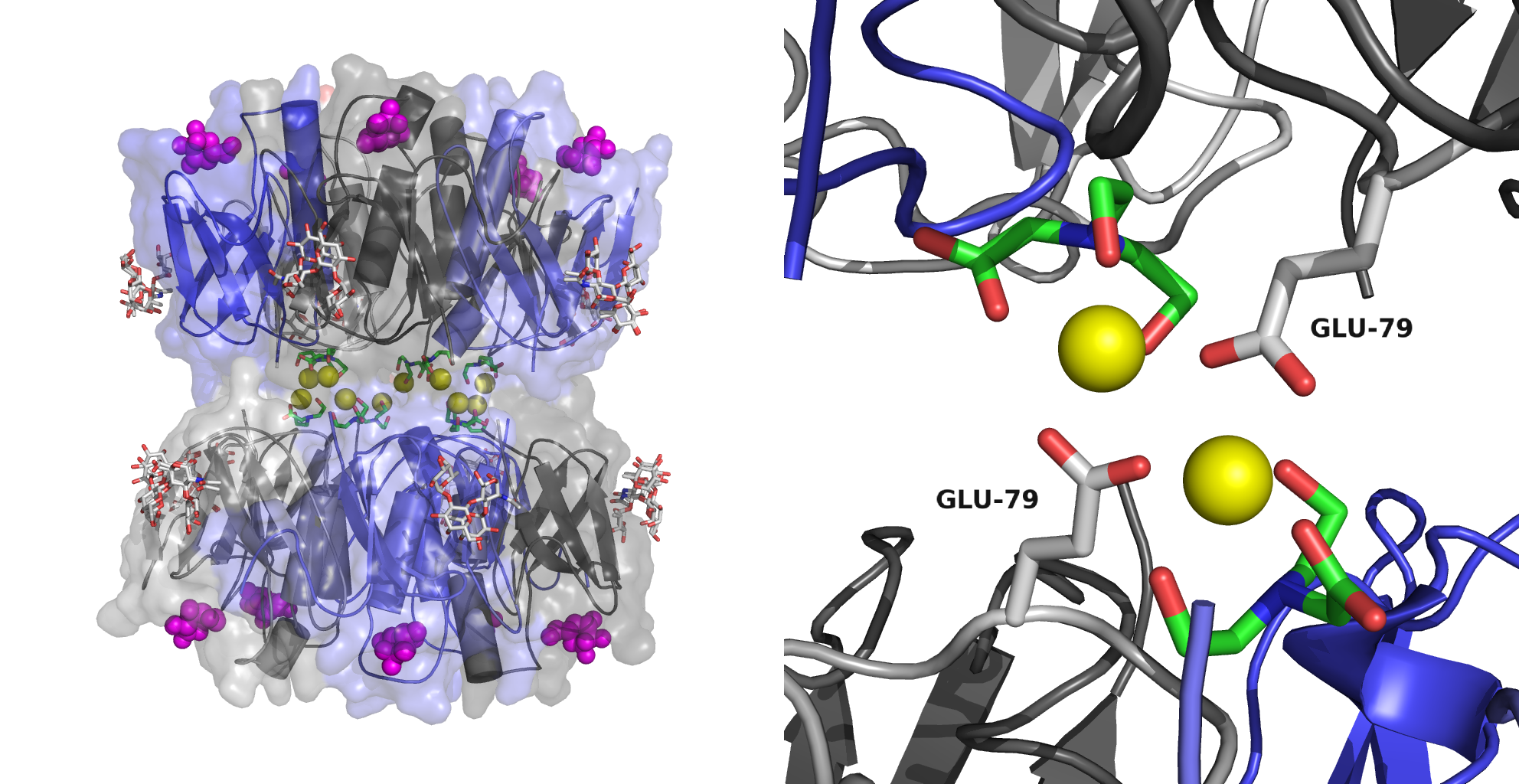

Supplement: S1 Fig — Left panel. Two cholera toxin B-pentamers positioned “top-to-top” in the asymmetric unit of cCTB in complex with H-tetra-BGA (PDB ID: 5ELB). H-tetra-BGA is depicted as white sticks, bicine as green sticks, calcium ions as yellow spheres and galactose molecules as magenta spheres. Buffer components facilitate the crystal contact. Right panel. Close-up view of the crystal contact between the two B-pentamers, with one glutamate residue from each B-pentamer and two bicine molecules (green) coordinating two calcium ions (yellow). (TIF) [file ppat.1005567.s002.tif]

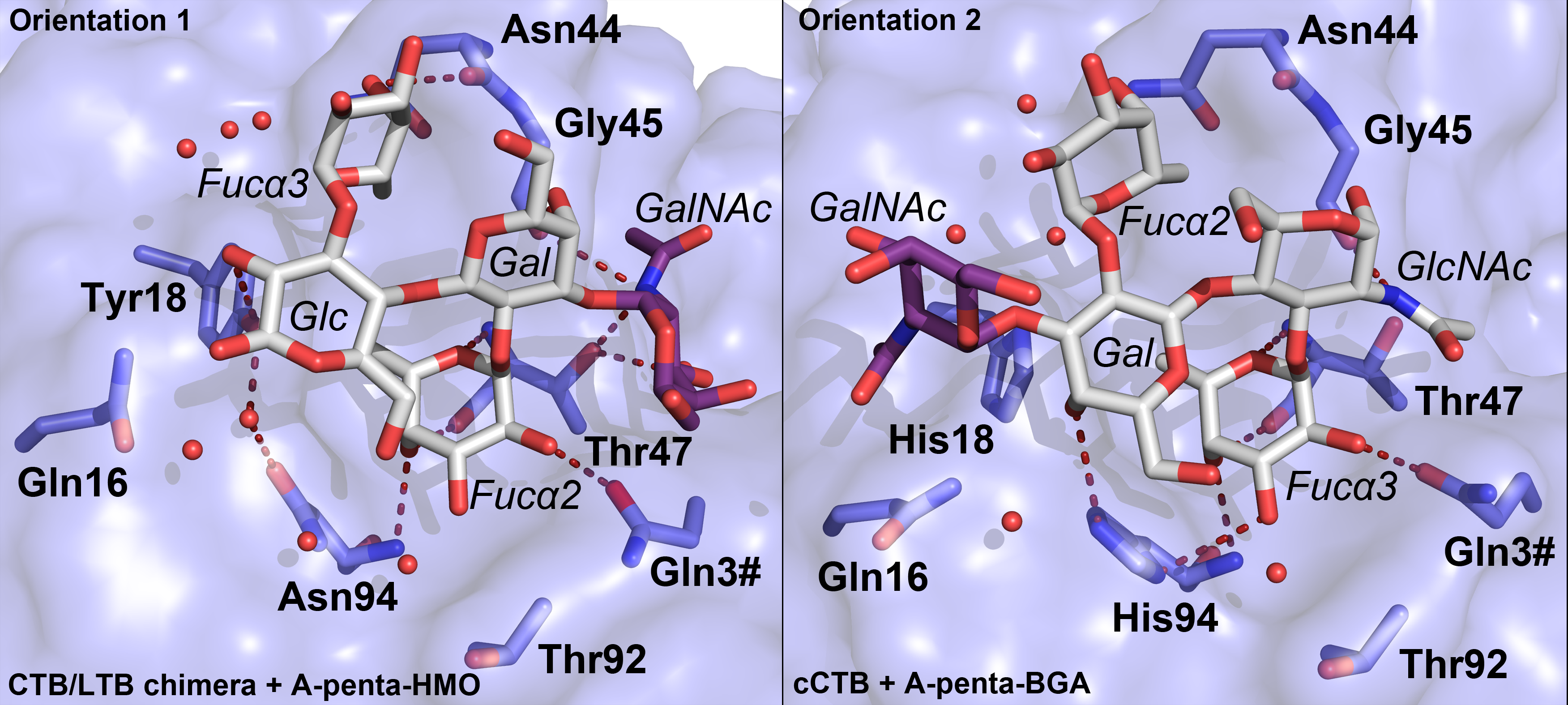

Supplement: S2 Fig — Left panel. Human milk oligosaccharide A-penta-HMO in complex with CTB/LTB chimera (PDB ID: 3EFX [26]; orientation 1). Right panel. Blood group A determinant A-penta-BGA in complex with cCTB (PDB ID: 5ELD; orientation 2). In both panels, the oligosaccharides are represented by white sticks, with the terminal GalNAc residue highlighted in purple. The relevant amino acid residues are shown as blue sticks, water molecules as red spheres, and hydrogen bonds as red dashed lines. Only hydrogen bonds conserved in most binding sites and with favorable angles are shown. (TIF) [file ppat.1005567.s003.tif]

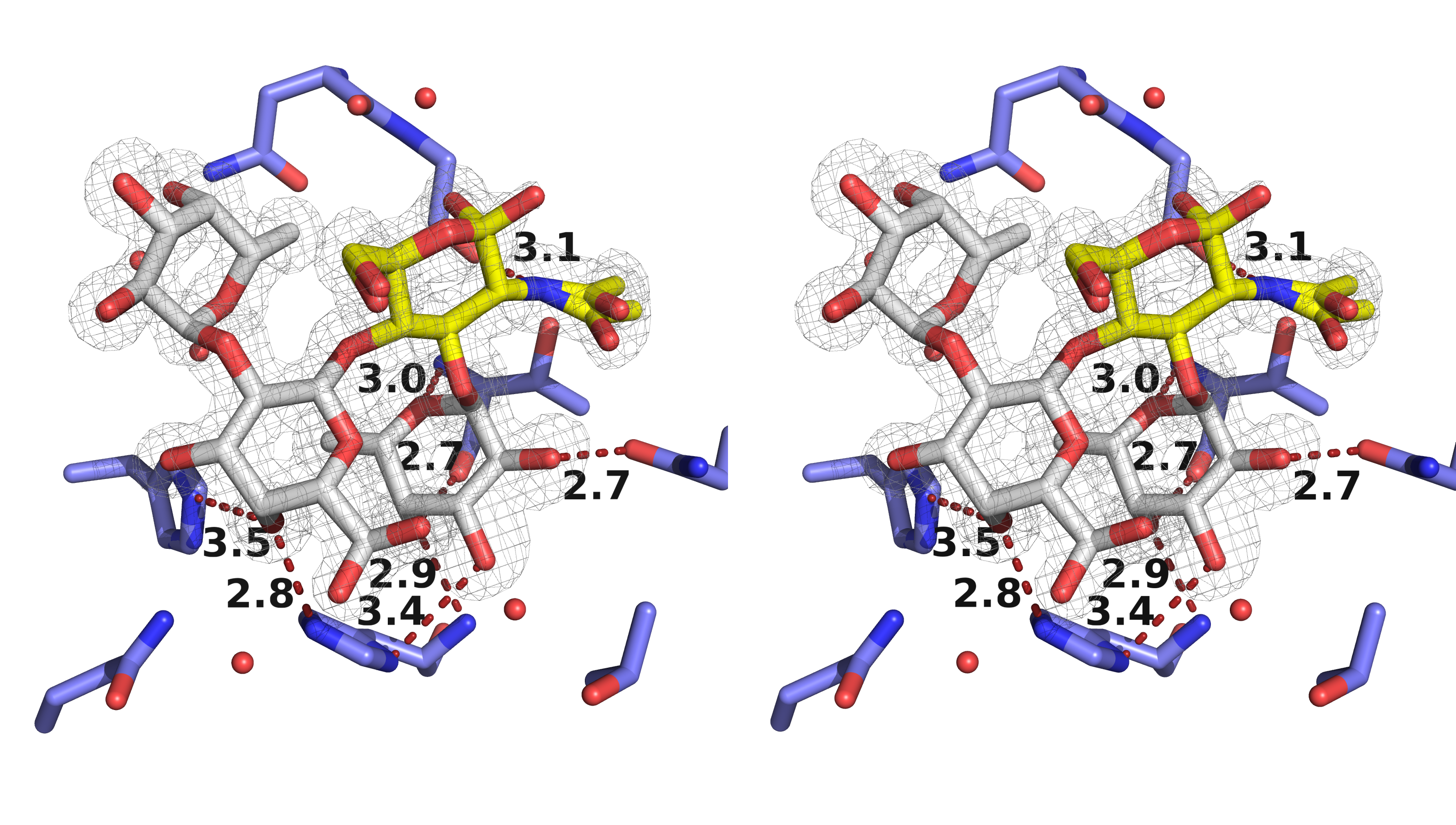

Supplement: S3 Fig — The oligosaccharide ligand is represented by white sticks, with the reducing-end GlcNAc residue highlighted in yellow. The relevant amino acid residues are shown as blue sticks. Water molecules are represented by red spheres, and hydrogen bonds by red dashed lines, with distances given in Ångström (PDB entry: 5ELB). The final σ A-weighted 2F o − F c electron density map is represented by a grey mesh and contoured at 1.0σ. (TIF) [file ppat.1005567.s004.tif]

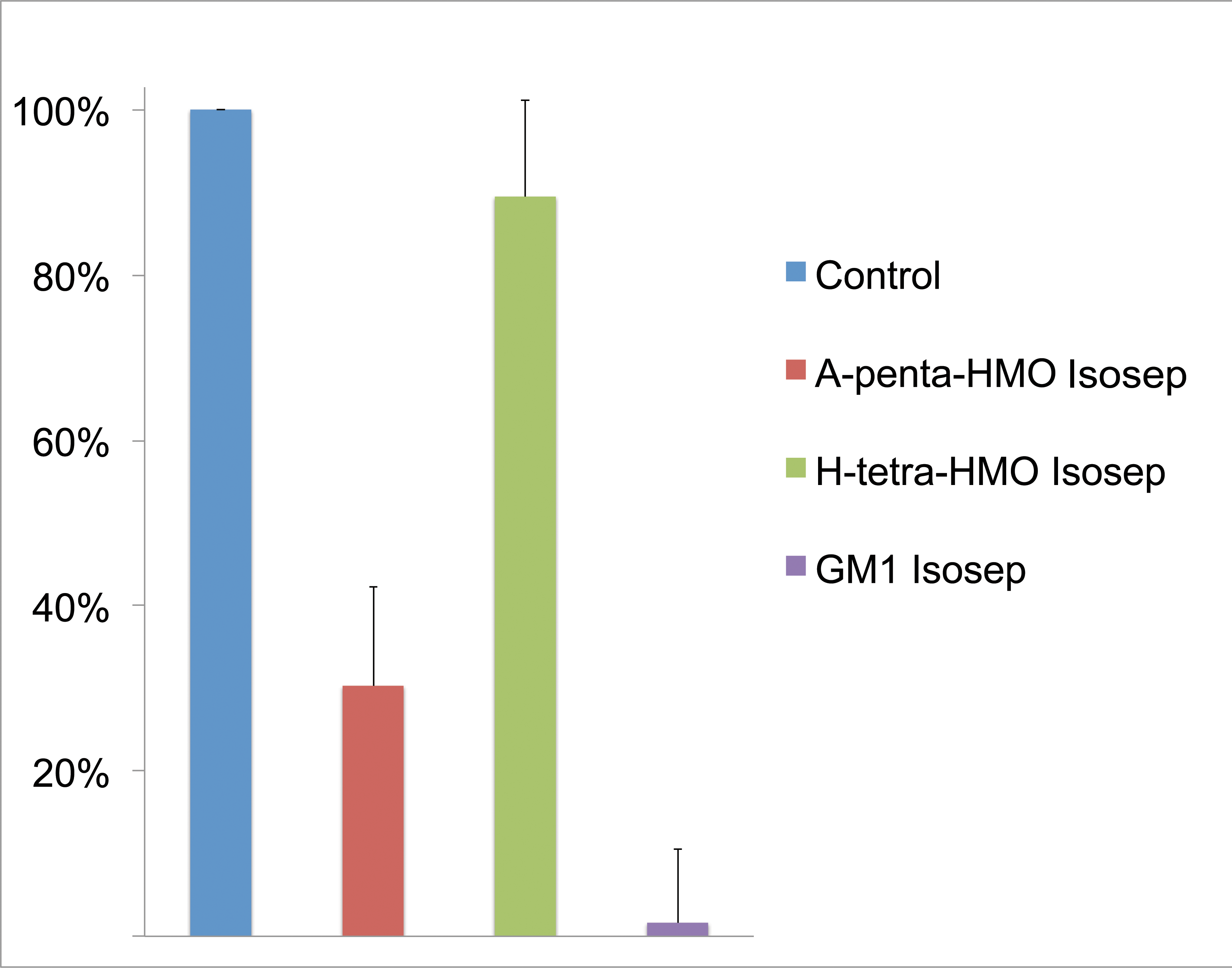

Supplement: S4 Fig — 100 ng/ml ET CTB was preincubated with 5 mM A-penta-HMO, 5 mM H-tetra-HMO or 5 μM GM1 ganglioside, and added to the GM1-coated wells. After washing, the remaining toxin was measured by absorbance at 405 nm. The y-axis shows percentage binding, with the control (ET CTB incubated with water) defined as 100%. All the ligands were purchased from Isosep AB. The experiments show that incubation of A-penta-HMO purchased from Isosep AB interfered with binding of CTB to GM1, and may hence contain trace amounts of GM1 or GM1 fragments. (TIF) [file ppat.1005567.s005.tif]
