# Supplementary material for: High-Resolution Crystal Structures Elucidate the Molecular Basis of Cholera Blood Group Dependence
Source: PLoS Pathog. 2016 Apr 15;12(4):e1005567. doi: 10.1371/journal.ppat.1005567 (PMC4833353; doi:10.1371/journal.ppat.1005567)
Supplement: S1 Table — (DOCX) [file ppat.1005567.s006.docx]

| **S1 Table.** **Carbohydrate-protein interactions** | | | | | |
| --- | --- | --- | --- | --- | --- |
| **cCTB + A-penta-BGA** | | | **ET CTB + A-penta-BGA** | | |
| Ligand residue | Direct contact (Å) | Interaction partner | Ligand residue | Direct contact (Å) | Interaction partner |
| GlcNAc O1 | 3.4 ± 0.0 (4/5) | Gly45 O | GlcNAc O1 | 3.5 ± 0.1 (3/10) | Gly45 O |
| GlcNAc N2 | 3.1 ± 0.1 (4/5) | Gly45 O | GlcNAc N2 | 2.8 ± 0.1 (3/10) | Gly45 O |
| Gal O4 | 3.5 ± 0.2 (4/5) | His18 Nε | Gal O4 | 2.6 ± 0.1 (3/10) | Tyr18 Oη |
|  | 3.3 ± 0.1 (4/5) | His94 Nε |  | 2.9 ± 0.1 (3/10) | His94 Nε |
|  | 3.4 ± 0.1 (4/5) | W1 |  | 3.5 ± 0.0 (1/10) | W1 |
| Fucα3 O2 | 2.6 ± 0.1 (4/5) | Gln3# Oε/Nε | Fucα3 O2 | 2.6 ± 0.1 (3/10) | Gln3# Oε/Nε |
| Fucα3 O3 | 3.4 ± 0.0 (4/5) | His94 N | Fucα3 O3 | 2.9 ± 0.0 (1/10) | His94 N |
|  | 2.7 ± 0.1 (4/5) | W2 |  | 2.9 ± 0.0 (1/10) | W2 |
| Fucα3 O4 | 3.0 ± 0.0 (4/5) | His94 N | Fucα3 O4 | 2.9 ± 0.1 (3/10) | His94 N |
|  | 2.6 ± 0.0 (4/5) | Thr47 O |  | 2.6 ± 0.0 (3/10) | Ile47 O |
| Fucα3 O5 | 3.0 ± 0.0 (4/5) | Thr47 N | Fucα3 O5 | 3.0 ± 0.1 (3/10) | Ile47 N |
| Listed are polar interactions of max. 3.5 Å and with favorable angles for H-bonds. Number of binding sites in which the interaction is present is shown in parentheses. Residues from neighboring subunits are marked by a hash (#). Only water molecules (W) conserved in most binding sites are shown. | | | | | |
